# Synchronization of Blood Flow Velocity in the Anterior Humeral Circumflex Artery and Reduction in Night Pain After Arthroscopic Rotator Cuff Repair: A Case Report

**DOI:** 10.7759/cureus.24468

**Published:** 2022-04-25

**Authors:** Akihisa Watanabe, Hinako Katayama, Takahiro Machida, Takahiko Hirooka

**Affiliations:** 1 Rehabilitation, Machida Orthopaedics, Kochi, JPN; 2 Orthopaedics, Machida Orthopaedics, Kochi, JPN; 3 Orthopaedic Surgery, Onomichi Municipal Hospital, Hiroshima, JPN

**Keywords:** rotator cuff tear, power doppler, night pain, blood flow velocity, arthroscopic rotator cuff repair, anterior humeral circumflex artery

## Abstract

Rotator cuff tears are commonly associated with pain at rest and at night particularly if lying on the affected shoulder. This case describes a 54-year-old man who reported concerns of severe night pain in his left shoulder and had to undergo arthroscopic rotator cuff repair. The severity of night pain and blood flow velocity in the anterior humeral circumflex artery (AHCA) were measured over time. The patient reported his night pain as 10/10 on the numerical rating scale in the first week after surgery. In the fourth week, he rated his night pain as 6/10, and in the fifth week, his pain was <2/10. We measured blood flow velocity in the AHCA using a 3-11MHz color Doppler and power Doppler ultrasound (SONIMAGE HS2, Konica Minolta, Tokyo, Japan), and we calculated peak systolic velocity. The course of peak systolic velocity in the AHCA ranged from 27.7 to 62.4 cm/s until four weeks after surgery when his night pain was severe; AHCA flow velocity ranged from 16.7 to 19.3 cm/s five weeks after surgery when his night pain had reduced. The initially high blood flow velocity in the AHCA decreased almost simultaneously with the improvement in night pain. Our case highlights that blood flow velocity in the AHCA synchronized with the severity of night pain, which may contribute to the understanding of sleep disturbances in patients after arthroscopic rotator cuff repair.

## Introduction

Rotator cuff tears (RCTs) typically cause shoulder pain at night [1]. The cause of night pain is multifactorial, including changes in skin temperature [2,3], subacromial pressure [4,5], synovitis [6], and blood flow velocity in the anterior humeral circumflex artery (AHCA) [7]. Other important causes of persistent night pain include a weak musculature around the arm and ineffective rehabilitation.

Terabayashi et al. conducted a recent cross-sectional study using power Doppler ultrasonography [7]. They found that patients with night pain had increased blood flow velocity in the AHCA compared with those without, suggesting that increased blood flow velocity reflects selective and pathognomonic changes in synovitis [7]. However, it is still unclear whether these results can be generalized to patients after arthroscopic rotator cuff repair (ARCR), and no longitudinal study has investigated this concept. Therefore, it is also unclear whether the increased blood flow velocity during severe night pain decreases synchronously with the improvement of night pain. Elucidation of these topics will help physicians better understand night pain and sleep disturbances in patients and improve treatment strategies and prognosis.

We report a case of a 54-year-old man with severe night pain in his left shoulder who underwent ARCR whose blood flow velocity in the AHCA correlated with the severity of his night pain. As this case highlights, the previous findings that AHCA blood flow velocity is associated with night pain [7] may be generalizable to patients after ARCR.

## Case presentation

A 54-year-old man reported concerns of pain in his left shoulder after a fall. He had no resting pain during the day but had to sleep sitting up because of pain at night. Despite four months of conservative treatment (i.e., analgesics and rehabilitation), his night pain did not improve. Magnetic resonance imaging showed an RCT with a Goutallier Grade 0, and he had to undergo ARCR. Preoperatively, his night pain was 8/10 on the 11-point numerical rating scale (NRS; 0 = no pain, 10 = worst pain imaginable), and his sleep disturbance was a Pittsburgh Sleep Quality Index (PSQI) score of 6 (PSQI of 5.5 or higher indicates poor sleep) [8]. No other diseases are suspected to be associated with night pain.

**Figure 1:**
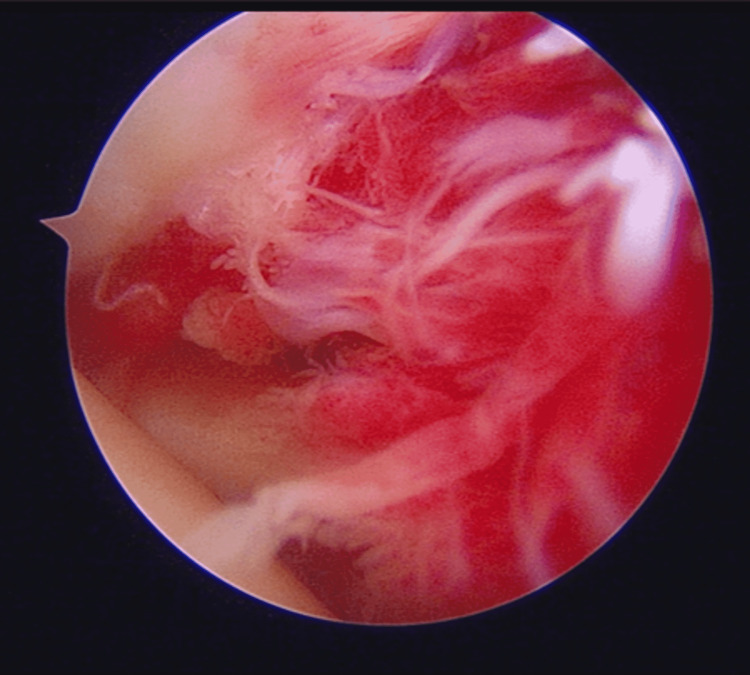
Arthroscopic view Arthroscopic intra-articular confirmation of synovitis of the lateral portion of the glenohumeral joint capsule.

The ARCR was performed in the beach chair position. The tear was a moderate crescent-shaped tear repaired according to the double anchor footprint fixation technique. Arthroscopy showed severe synovitis of the lateral portion of the glenohumeral joint capsule with a Davis score of 5 (the capsule was red, the villous projections were extensive, and the capillaries were hypertrophied; Figure 1) [9].

After ARCR, the shoulder was immobilized in an abduction brace for six weeks, and the patient was admitted to the clinic for the entire duration of wearing the brace. He underwent standard rehabilitation practices recommended by the American Society of Shoulder and Elbow Therapists. He started passive movement exercises two weeks after ARCR, and active movement exercises six weeks after ARCR. Analgesic drugs were used as appropriate, and non-steroidal anti-inflammatory drugs (NSAIDs, celecoxib), paracetamol, and tramadol hydrochloride were prescribed (Table 1).

**Table 1:** Course of pain, sleep disturbance, blood flow velocity, and medication Abbreviations: NRS, 11-point numerical rating scale; PSQI, Pittsburgh Sleep Quality Index; PCS, Pain Catastrophizing Scale; HADS-A, Hospital Anxiety and Depression Scale -Anxiety; HADS-D, Hospital Anxiety and Depression Scale -Depression; PSV, peak systolic velocity in the anterior humeral circumflex artery; n/a, not available.

	Presurgery	Postsurgery, week					
		1	2	3	4	5	6
Pain during the day, NRS	0	6	0	0	0	0	0
Pain at night, NRS	8	10	6	6	6	2	2
PSQI	6	14	14	14	13	12	5
PCS	n/a	n/a	15	n/a	n/a	n/a	n/a
HADS-A	n/a	n/a	6	n/a	n/a	n/a	n/a
HADS-D	n/a	n/a	5	n/a	n/a	n/a	n/a
PSV, cm/s	62.4	n/a	29.6	27.7	28.4	16.7	19.3
Medication, mg/day							
Celecoxib	0	200	200	200	200	200	200
Tramadol Hydrochloride	37.5	37.5	87.5	137.5	137.5	137.5	137.5
Paracetamol	325	325	325	325	325	325	325

**Figure 2:**
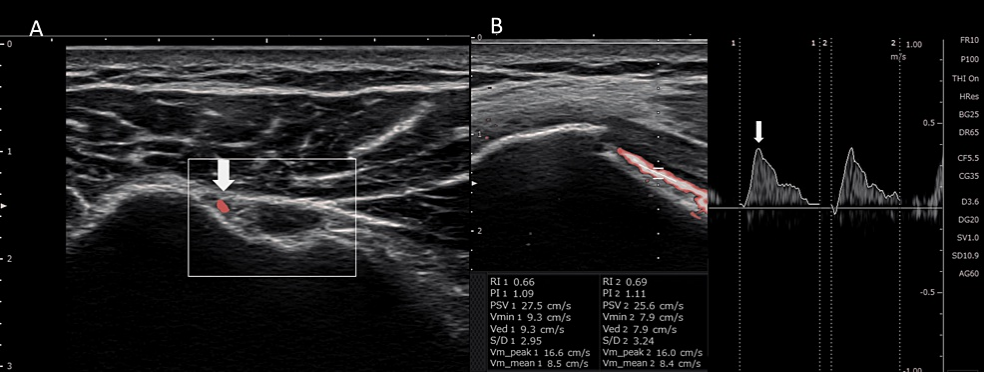
Ultrasonography A) Transverse color Doppler image of the bicipital groove with the anterior humeral circumflex artery (arrow). B) Longitudinal-pulse Doppler image of the anterior humeral circumflex artery with peak systolic velocity (arrow).

We measured his blood flow velocity in the AHCA with a 3-11MHz color Doppler and powered Doppler ultrasound (SONIMAGE HS2, Konica Minolta, Tokyo, Japan), and we calculated his peak systolic velocity (PSV) according to the method reported by Terabayashi et al. [7]. Briefly, the patient was resting in a chair for five minutes with the elbow joint bent at 90 degrees, the shoulder joint abducted at 30 degrees, and the hand placed palm-up on the armrest. First, we identified the bicipital groove and AHCA in a transverse scanning (Figure 2A). Next, the PSV in the AHCA was measured in a longitudinal scan (Figure 2B). The measurements were taken three times, and mean values were calculated. High reliability has been reported for this method (intraobserver reproducibility, 0.983; interobserver reproducibility, 0.949) [7].

The patient's night pain was NRS 10/10 in the first week after ARCR and NRS 6/10 in the fourth week. At this stage, he had no pain in the daytime, and his night pain interfered with sleep and made him want to sit up. Five weeks after ARCR, he could finally sleep soundly in the supine position and reported the night pain had reduced to NRS 2/10. The course of PSVs in the AHCA ranged from 27.7 to 62.4 cm/s four weeks after the ARCR when his night pain was severe and from 16.7 to 19.3 cm/s five weeks after the ARCR when night pain was reduced. The night pain and sleep disturbances during this period are shown in Table 1. In brief, the reduction of night pain and decreased PSV occurred almost simultaneously. Sleep disturbances also improved to PSQI 5 at six weeks after the ARCR. No psychological factors were noted. After that, the absence of night pain and sleep disturbance persisted, and rehabilitation was continued. His function improved sufficiently six months after the ARCR. No serious adverse events were reported during this study period.

## Discussion

Our patient's blood flow velocity in the AHCA was elevated in the preoperative and early postoperative periods when the patient was in severe pain. Tada et al. calculated a PSV cutoff value of 20.5 cm/s to determine the presence of night pain (Table 2) [10]. The PSVs in our patient during his severe pain were always above the cutoff value (range, 27.7 to 62.4 cm/s). Furthermore, arthroscopic examination revealed severe synovitis in the lateral portion of the glenohumeral joint capsule. The lateral portion of the glenohumeral joint capsule receives its blood supply from the AHCA [11], and increased blood flow velocity in the AHCA has been reported to reflect synovitis in this area [7]. Recent studies have indicated that severe synovitis in the glenohumeral joint may be involved in the pathogenesis of pain [12,13]. Thus, our finding that blood flow velocity, which may reflect synovitis, was increased during the severe night pain phase in the patient after ARCR coincides with a recent study on RCT patients [7].

**Table 2:** Summary of recent studies and current case report characteristics Abbreviations: night pain (+), patients with rotator cuff tears with night pain; night pain (-), patients with rotator cuff tears without night pain; PSV, peak systolic velocity in the anterior humeral circumflex artery (cm/s); n/a, not available. Terabayashi et al., 2014 [7]; Tada et al., 2015 [10]

Authors, year	Night Pain (+)		Night Pain (-)		Cutoff	Odds Ratio
	n	PSV cm/s, mean (SD)	n	PSV cm/s, mean (SD)		
Terabayashi et al., 2014	34	34.9 (16.5)	13	22.6 (10.7)	n/a	n/a
Tada et al., 2015	n/a	31.7 (14.1)	n/a	19.7 (9.6)	20.5	30.9
Current case report	-	Range: 27.7 to 62.4	-	Range: 16.7 to 19.3	-	-

The reduction in night pain and blood flow velocity in the AHCA occurred almost simultaneously. The PSVs in this patient finally fell below the cutoff value of 20.5 cm/s when the night pain was reduced (range, 16.7 to 19.3 cm/s). Although the severity of synovitis during this period could not be confirmed by arthroscopy, the decrease in blood flow velocity may reflect a decrease in inflammation [7]. This is the first case report to demonstrate the synchronization of night pain and blood flow velocity in the AHCA by observing a decline from severe pain to pain relief.

Several authors have reported on other mechanisms of night pain in patients with RCT [2-6]. Modulation of shoulder skin temperature has been associated with night pain [2]. However, postoperative shoulder temperatures have been reported to be higher due to inflammation [3]. Intra-articular blood flow is probably affected by various factors, including synovitis [6]. Thus, changes in blood flow velocity in the AHCA can mediate synovitis and partially explain or predict changes in night pain. Increased subacromial pressure has also been described as a cause of night pain, and this pressure varies with sleep position [4,5]. During the research period, this patient's affected limb was immobilized with a brace during the day, and his sleeping posture was always supine. In this case, confounding external factors such as excessive shoulder movement during the day or sleeping posture have been eliminated.

The blood flow velocity and sleep disturbances improved synchronously with night pain. However, sleep disturbances can be caused by multiple factors and night pain in the shoulder, such as poor mental health, psychiatric disorders, and depression [1]. Furthermore, despite the absence of significant pain, postoperative patients reported suffering from severe sleep disturbances [14]. We used the Pain Catastrophizing Scale and the Hospital Anxiety and Depression Scale to assess our patient's psychological factors, and the results were unremarkable. In this patient, blood flow velocity may have affected sleep disturbance mediated by night pain rather than psychological factors. Since sleep quality has been highlighted as a health factor that should be noted for patients undergoing ARCR [15], these results will contribute to understanding sleep disturbances in patients after ARCR.

Our findings that night pain was synchronous with blood flow velocity, which may reflect synovitis, suggest that NSAIDs are candidates for night pain management. Although NSAIDs have been reported to increase rotator cuff retear [16], their impact on retear is limited and controversial [17]. Risk factors for rotator cuff retear include age or anatomical factors such as tear size and fatty infiltration [18], which did not apply to this patient. Therefore, we decided that the benefit of the anti-inflammatory effect of NSAIDs outweighed the harm that NSAIDs may cause in terms of rotator cuff retear. This patient's multimodal administration of celecoxib, paracetamol, and tramadol [19] resulted in early relief of severe pain and sleep disturbance, and the anti-inflammatory effect was indirectly confirmed by blood flow velocity. Six weeks after the ARCR, our patient's PSQI score was 5, much better than in previously reported results six weeks after surgery [15,20].

Our patient's pain interfered with his sleep and made him want to sit up, the same inclusion criteria for Terabayashi et al.'s study [7]. Therefore, when generalizing the findings from this case, it is important to discern the different types of night pain a patient experiences, such as pain on turning over or pressure pain on the affected side in the side-lying position, before generalizing the findings from this case.

## Conclusions

The initially high blood flow velocity in the AHCA decreased almost simultaneously with the improvement in night pain and sleep disturbance. Therefore, blood flow velocity in the AHCA can be a parameter reflecting the presence of night pain. Future studies focusing on the blood flow velocity in the AHCA with a larger sample size, including randomized controlled trials, will help form a better understanding and management of patients with night pain.
